# The prevalence of child maltreatment in Australia: findings from a national survey

**DOI:** 10.5694/mja2.51873

**Published:** 2023-04-02

**Authors:** Ben Mathews, Rosana Pacella, James G Scott, David Finkelhor, Franziska Meinck, Daryl J Higgins, Holly E Erskine, Hannah J Thomas, David M Lawrence, Divna M Haslam, Eva Malacova, Michael P Dunne

**Affiliations:** ^1^ Queensland University of Technology Brisbane QLD; ^2^ Bloomberg School of Public Health Johns Hopkins University Baltimore MD United States of America; ^3^ Institute for Lifecourse Development University of Greenwich London United Kingdom; ^4^ Child Health Research Centre, the University of Queensland Brisbane QLD; ^5^ QIMR Berghofer Medical Research Institute Brisbane QLD; ^6^ Crimes against Children Research Center University of New Hampshire Durham NH United States of America; ^7^ University of Edinburgh Edinburgh United Kingdom; ^8^ University of the Witwatersrand Johannesburg Johannesburg South Africa; ^9^ Institute of Child Protection Studies Australian Catholic University Melbourne VIC; ^10^ The University of Queensland Brisbane QLD; ^11^ Queensland Centre for Mental Health Research Brisbane QLD; ^12^ Curtin University Perth WA; ^13^ Parenting and Family Support Centre the University of Queensland Brisbane QLD; ^14^ Institute for Community Health Research Hue University Hue City Vietnam

**Keywords:** Child welfare, Child health, Adolescence, Child abuse, Epidemiology

## Abstract

**Objectives:**

To estimate the prevalence in Australia of each type of child maltreatment; to identify gender‐ and age group‐related differences in prevalence.

**Design, setting:**

Cross‐sectional national survey; mobile telephone interviews using random digit dialling (computer‐generated), Australia, 9 April – 11 October 2021. Retrospective self‐report data using validated questionnaire (Juvenile Victimisation Questionnaire‐R2 Adapted Version (Australian Child Maltreatment Study).

**Participants:**

People aged 16 years or more. The target sample size was 8500 respondents: 3500 people aged 16–24 years and 1000 respondents each from five further age groups (25–34, 35–44, 45–54, 55–64, 65 years or more).

**Main outcome measures:**

Proportions of respondents reporting physical abuse, sexual abuse, emotional abuse, neglect, and exposure to domestic violence to age 18 years, assessed with the Juvenile Victimization Questionnaire‐R2 Adapted Version (Australian Child Maltreatment Study), overall and by gender and age group, and weighted to reflect characteristics of the Australian population aged 16 years or more in 2016.

**Results:**

Complete survey data were available for 8503 eligible participants (14% response rate). Physical abuse was reported by 32.0% of respondents (95% confidence interval [CI], 30.7–33.3%), sexual abuse by 28.5% (95% CI, 27.3–29.8%), emotional abuse by 30.9% (95% CI, 29.7–32.2%), neglect by 8.9% (95% CI, 8.1–9.7%), and exposure to domestic violence by 39.6% (95% CI, 38.3–40.9%). The proportions of respondents who reported sexual abuse, emotional abuse, or neglect were each statistically significantly larger for women than men. The reported prevalence of physical abuse by respondents aged 16–24 years was lower than for those aged 25–34 years, and that of sexual abuse was lower than for those aged 35–44 years, suggesting recent declines in the prevalence of these maltreatment types.

**Conclusions:**

Child maltreatment is common in Australia, and larger proportions of women than men report having experienced sexual abuse, emotional abuse, and neglect during childhood. As physical and sexual abuse may have declined recently, public health policy and practice may have positive effects, justifying continued monitoring and prevention activities.

**The known:** Child maltreatment — physical abuse, sexual abuse, emotional abuse, neglect, and exposure to domestic violence — is a major public health problem. Population‐level evidence regarding the prevalence of child maltreatment in Australia is not available.

**The new:** Physical abuse, sexual abuse, emotional abuse, and exposure to domestic violence were frequently reported by survey respondents, and women reported sexual abuse, emotional abuse, and neglect more often than men. Recent declines in physical and sexual abuse may reflect the effect of public health interventions.

**The implications:** Our findings are relevant to the health, education, and welfare sectors. Public health policy and prevention efforts can further reduce levels of physical and sexual abuse, and reduce those of emotional abuse and exposure to domestic violence.

Child maltreatment — physical abuse, sexual abuse, emotional abuse, neglect, and exposure to domestic violence — is a major public health challenge. Systematic reviews and meta‐analyses have found that child maltreatment is associated with mental health disorders, physical health problems, and health risk behaviours throughout life.^1^, ^2^, ^3^ The magnitude of the challenge to individuals and to society is reflected in the United Nations’ Sustainable Development Goals Target 16.2, which aims to end all forms of violence against children.^4^

Public health efforts to prevent, identify, and respond to child maltreatment must be informed by reliable evidence about its prevalence, nature, and associated health effects. However, rigorous evidence about its prevalence is scarce, both in Australia and overseas. Meta‐analyses have generated global estimates of the prevalence of specific maltreatment types (based on self‐reports): physical abuse (22.6%),^5^ sexual abuse (12.7%),^6^ and emotional abuse (36.3%).^7^ In a recent systematic review, we found that only three national studies had assessed the prevalence of all five major types of child maltreatment to age 18 years in a representative population sample,^8^ including a United Kingdom study (physical abuse, 8.4%; sexual abuse, 24.1%; emotional abuse, 6.9%; neglect, 16%; exposure to domestic violence, 23.7%).^9^

The prevalence of all five types of maltreatment has not been assessed in a nationally representative Australian sample.^8^, ^10^ A 2000 survey of a nationally representative sample of 1784 adults aged 18–59 years found that 33.6% of women and 15.9% of men reported non‐penetrative sexual abuse before the age of 16; 12% of women and 4% of men reported penetrative sexual abuse.^11^ Similarly, cohort studies and personal safety surveys have examined selected aspects of child maltreatment, but have not comprehensively investigated its prevalence or nature.^12^, ^13^, ^14^, ^15^

Defining the epidemiology of child maltreatment in Australia requires rigorous approaches informed by the best relevant studies, a large, nationally representative sample, and assessment of all types of self‐reported maltreatment to the age of 18 years. Assessing whether these experiences were isolated or repeated is also important.

One primary aim of the Australian Child Maltreatment Study (ACMS) was to determine the prevalence of each of the five types of child maltreatment in Australia. The ACMS also determined prevalence by gender and age group.

## Methods

The ACMS is a cross‐sectional survey study of people in Australia aged 16 years or more about their childhood and health. As detailed elsewhere in this supplement,^16^ participants were recruited using a mobile phone sampling frame and random digit dial methodology.

### Measures

Child maltreatment was assessed with the Juvenile Victimization Questionnaire (JVQ)‐R2: Adapted Version (Australian Child Maltreatment Study),^17^ an adapted and validated version of an instrument used in national studies in the United States,^18^ the United Kingdom,^9^ and other countries,^8^, ^19^, ^20^ as well as in smaller studies.^21^ The adaptation and validation of the instrument is described in detail elsewhere.^22^

The adapted version of the JVQ‐R2 includes 16 items that assessed all five major types of maltreatment.^16^ The behaviourally specific questions elicited dichotomous responses (yes or no) about whether the participant had experienced any subdomain of each maltreatment type (physical abuse, two subdomains; sexual abuse, four; emotional abuse, three; neglect, three; exposure to domestic violence, four subdomains). Follow‐up questions assessed characteristics of these experiences, including the number of times they were experienced. The survey also included items on other adverse experiences during childhood, peer bullying, mental health disorders, physical health, health risk behaviours, and health service use.

### Definitions

As described in the protocol,^17^ we applied definitions of each type of child maltreatment that were based on rigorous conceptual models.^23^, ^24^, ^25^, ^26^, ^27^ Accordingly, physical abuse, emotional abuse, neglect, and exposure to domestic violence were conceptualised as acts and omissions by parents and parent‐like caregivers, while sexual abuse included acts by any person. Informed by these models, and by previous studies using the original Juvenile Victimization Questionnaire, we designed questions that reflected operational definitions of each maltreatment type.

### Procedures

The study procedures are detailed elsewhere in this Supplement.^16^ In short, the target survey respondent number was 8500: 3500 people aged 16–24 years (purposively oversampled) and 1000 in each of the age groups 25–34, 35–44, 45–54, 55–64, and 65 years or more. To ensure that the sample was representative of the Australian population, survey data were weighted by age group, gender, Indigenous status, country of birth (Australia or overseas), highest educational level, and residential socio‐economic status (Relative Socio‐economic Advantage and Disadvantage quintiles).^16^

### Statistical analysis

Data from the computer‐assisted telephone interview software platform were imported into SAS 9.4; data were cleaned by author DL and a research assistant. We calculated prevalence rates for the whole sample, and by gender and age group, applying methods based on conceptual models^23^, ^24^, ^25^, ^26^, ^27^ and employed by other studies.^9^, ^18^, ^19^, ^20^, ^28^ For physical abuse, sexual abuse, and exposure to domestic violence, prevalence was based on affirmative responses to any question on these maltreatment types, regardless of frequency. For emotional abuse and for neglect, we estimated prevalence from affirmative responses to any of the questions if experienced over a period of weeks, months, or years.^23^, ^24^, ^28^ For sexual abuse, we separately estimated rates of abuse by any person and by adult family members.^18^ The prevalence of contact sexual abuse was estimated from affirmative responses to one or more of three contact sexual abuse questions — contact abuse without intercourse (touching); attempted forced intercourse; forced intercourse — and the prevalence of non‐contact sexual abuse was estimated from affirmative responses to the sole sexual abuse question on exposure or voyeurism.

The frequency of maltreatment is rarely reported, and has been assessed with different methods.^29^ We estimated frequency by categorising the number of times the experiences were reported, consistent with other studies in Australia^30^, ^31^ and Canada.^25^

Survey‐weighted data for the five child maltreatment types were summarised as numbers and proportions with 95% confidence intervals (CIs) calculated using the Taylor series method. The statistical significance of differences in proportions by gender or age group were assessed in Rao–Scott χ^2^ tests. All analyses were conducted in SAS version 9.4; graphs were prepared in Stata 17. Each analysis was independently checked by two co‐authors, in random spot checks of SAS code and checking of analysis results in SPSS 28.

### Ethics approval

The Queensland University of Technology Human Research Ethics Committee approved the study (#1900000477). Each survey respondent provided verbal informed consent to participation.

## Results

We estimated that 210 373 of the 404 180 people we attempted to contact would have been eligible to participate in our survey; contact was made with 60 803, of whom 8503 completed the survey. The response rate with respect to the estimated number of eligible candidates was 4.0%; with respect to eligible candidates contacted it was 14.0%. The demographic characteristics of the sample (apart from age distribution) broadly matched those for people aged 16 years or more in the 2016 Australian census. Potential participation biases were deemed to be minor.^16^

### Prevalence of child maltreatment types

A total of 2623 respondents reported physical abuse during childhood (32.0%; 95% CI, 30.7–33.3%) (Box 1). The subdomains of severe (1507 people, 18.3%) and moderate physical abuse (1639, 20.1%) were reported by similar proportions of respondents (Supporting Information, table 1). Emotional abuse was reported by 2743 people (30.9%; 95% CI, 29.7–32.2%) (Box 1). Subdomains of emotional abuse were reported at different rates: hostile interactions were reported by 2154 (23.8%), rejection by 810 (8.8%), and emotional unavailability by 1870 people (21.6%) (Supporting Information, table 2). Neglect was reported by 759 people (8.9%; 95% CI, 8.1–9.7%) (Box 1). Subdomains of neglect were reported at different rates: environmental neglect was reported by 361 (3.9%), physical neglect by 335 (4.1%), and medical neglect by 402 people (4.8%) (Supporting Information, table 3). Exposure to domestic violence was reported by 3487 people (39.6%; 95% CI, 38.3–40.9%) (Box 1). Subdomains of domestic violence were reported at different rates: exposure to inter‐parental physical violence was reported by 1606 (19.9%), serious threats by 1310 (15.5%), parents damaging property by 2612 (28.7%), and inter‐parental intimidation or control by 1921 people (22.0%) (Supporting Information, table 4).

Box 1 Weighted proportions (with 95% confidence intervals) of survey respondents who reported maltreatment during childhood, by maltreatment type, age group, and gender*

**Table:** 

Age group	Number of respondents	Physical abuse	Sexual abuse	Emotional abuse	Neglect	Exposure to domestic violence
All ages	8503	32.0% (30.7–33.3%)	28.5% (27.3–29.8%)	30.9% (29.7–32.2%)	8.9% (8.1–9.7%)	39.6% (38.3–40.9%)
Women	4182	31.5% (29.7–33.3%)	37.3% (35.5–39.2%)^†^	35.6% (33.8–37.4%)^†^	10.8% (9.5–12.0%)^†^	40.8% (38.9–42.6%)
Men	4195	32.1% (30.3–33.9%)	18.8% (17.3–20.3%)	25.4% (23.7–27.1%)	6.7% (5.7–7.6%)	38.0% (36.1–39.9%)
Gender‐diverse	126	50% (37–63%)	52% (39–65%)	58% (45–71%)	26% (15–38%)	58% (45–71%)
16–24 years	3500	28.2% (26.6–29.9%)	25.7% (24.1–27.3%)	34.6% (32.8–36.3%)	10.3% (9.2–11.4%)	43.8% (42.0–45.6%)
Women	1662	29.0% (26.6–31.4%)	35.2% (32.7–37.8%)^†^	40.5% (37.9–43.1%)^†^	12.5% (10.7–14.2%)^†^	45.8% (43.2–48.5%)
Men	1748	26.3% (24.0–28.5%)	14.5% (12.6–16.5%)	26.9% (24.6–29.3%)	7.2% (5.8–8.5%)	40.8% (38.2–43.3%)
25–34 years	1000	36.0% (32.7–39.3%)^‡^	27.4% (24.4–30.5%)	36.7% (33.5–40.0%)	11.7% (9.5–14.0%)	48.1% (44.7–51.5%)
Women	470	37.7% (32.9–42.5%)	37.6% (32.8–42.4%)^†^	42.7% (37.9–47.6%)^†^	15% (11–18%)	49.0% (44.1–54.0%)
Men	516	34.0% (29.5–38.5%)	16.9% (13.4–20.5%)	29.8% (25.4–34.1%)	8.3% (5.6–11%)	47.0% (42.2–51.7%)
35–44 years	1000	33.2% (30.0–36.5%)^‡^	30.3% (27.1–33.5%)^‡^	31.2% (28.0–34.4%)	9.3% (7.2–11%)	43.9% (40.4–47.3%)
Women	516	33.3% (28.8–37.9%)	40.1% (35.3–44.8%)^†^	37.8% (33.2–42.5%)^†^	13% (9.3–16%)^†^	45.7% (40.9–50.5%)
Men	476	32.8% (28.1–37.6%)	20% (16–24%)	24.1% (19.7–28.5%)	5.7% (3.3–8.0%)	41.5% (36.5–46.5%)
45–54 years	1002	34.2% (30.9–37.5%)^‡^	29.8% (26.7–32.9%)	33.0% (29.7–36.2%)	7.5% (5.5–9.4%)	41.3% (38.0–44.7%)
Women	521	32.8% (28.2–37.3%)	38.8% (34.1–43.5%)^†^	37.5% (32.9–42.2%)	8.1% (5.3–11%)	42.4% (37.6–47.1%)
Men	477	36.0% (31.2–40.7%)	20.5% (16.6–24.5%)	28.6% (24.1–33.1%)	6.8% (4.1–9.4%)	40.1% (35.2–45.0%)
55–64 years	1001	35.2% (31.9–38.4%)^‡^	30.7% (27.6–33.9%)	32.7% (29.5–35.9%)	8.4% (6.5–10%)	39.1% (35.7–42.4%)
Women	509	34.4% (29.8–39.1%)	40.2% (35.5–45.0%)^†^	37.4% (32.7–42.1%)^†^	10% (7.1–13%)	39.9% (35.2–44.6%)
Men	487	35.5% (30.8–40.2%)	19.8% (16.1–23.6%)	27.6% (23.3–31.8%)	6.6% (4.2–9.0%)	38.2% (33.4–43.0%)
65 years or older	1000	26.0% (23.0–29.0%)	27.4% (24.3–30.5%)	20.5% (17.7–23.3%)^‡^	6.8% (5.0–8.6%)^‡^	25.0% (22.1–28.0%)^‡^
Women	504	24.1% (20.0–28.1%)	33.4% (28.9–37.9%)^†^	23.2% (19.2–27.3%)	7.6% (5.0–10%)	27.3% (23.1–31.5%)
Men	491	28.2% (23.6–32.7%)	20.1% (16.0–24.1%)	17% (13–21%)	5.7% (3.3–8.0%)	22.2% (18.1–26.4%)

CI = confidence interval.

* The raw numbers underlying the listed proportions are included in the Supporting Information, tables 1–5. Proportions are weighted by age group, gender, Indigenous status, country of birth (Australia or overseas), highest educational level, and residential socio‐economic status (Relative Socio‐economic Advantage and Disadvantage quintiles).^16^

† Confidence intervals for women and men do not overlap (ie, statistically significant difference in proportions).

‡ Confidence intervals for respondents aged 16–24 years and respective age group (combined men and women only) do not overlap (ie, statistically significant difference in proportions). The difference for sexual abuse between the 16–24‐ and 35‐44‐year groups was also deemed to be meaningfully different.

A total of 2348 respondents reported sexual abuse by any person (28.5%; 95% CI, 27.3–29.8%) (Box 1). Contact sexual abuse was reported by 1960 people (23.7%; 95% CI, 22.6–24.9%), including sexual touching by 1525 (18.9%), attempted forced intercourse by 1201 (13.8%), and forced intercourse by 717 (8.7%). Non‐contact sexual abuse was reported by 1443 people (18.1%; 95% CI, 17.0–19.1%) (Supporting Information, table 5).

Sexual abuse by an adult family member was reported by 551 people (7.8%; 95% CI, 7.0–8.5%). Contact sexual abuse by adult family members was reported by 464 people (6.7%; 95% CI, 6.0–7.5%), including sexual touching by 423 (6.1%), attempted forced intercourse by 214 (3.1%), and forced intercourse by 135 (1.9%) (Supporting Information, table 6).

### Prevalence of child maltreatment types, by gender

Physical abuse during childhood was reported by 1281 women (31.5%; 95% CI, 29.7–33.3%) and 1284 men (32.1%; 95% CI, 30.3–33.9%). Emotional abuse was reported by 1573 women (35.6%; 95% CI, 33.8–37.4%) and 1088 men (25.4%; 95% CI, 23.7–27.1%); neglect was reported by 449 women (10.8%; 95% CI, 9.5–12.0%) and 276 men (6.7%; 95% CI, 5.7–7.6%); exposure to domestic violence was reported by 1790 women (40.8%; 95% CI, 38.9–42.6%) and 1619 men (38.0%; 95% CI, 36.1–39.9%). For emotional abuse (exceptions: 45–54 years, 65 years or more) and neglect (exceptions: 45–54 years, 55–64 years, 65 years or more) the confidence intervals for the weighted proportions for men and women did not overlap, overall or by age group (Box 1; Supporting Information, tables 1–4).

Sexual abuse was reported by 1536 women (37.3%; 95% CI, 35.5–39.2%) and 739 men (18.8%; 95% CI, 17.3–20.3%) (Box 1; Supporting Information, table 5). Contact sexual abuse was reported by 1336 women (32.4%; 95% CI, 30.6–34.2%) and 563 men (14.2%; 95% CI, 12.9–15.6%), sexual abuse by an adult family member by 407 women (11.9%; 95% CI, 10.6–13.2%) and 122 men (3.2%; 95% CI, 2.5–3.9%), and contact sexual abuse by adult family members by 351 women (10.5%; 95% CI, 9.3–11.8%) and 97 men (2.6%; 95% CI, 1.9–3.2%) (Supporting Information, tables 5 and 6).

The proportions of the 126 gender‐diverse respondents (ninety aged 16–24 years) who reported each type of maltreatment were larger than for other respondents (Box 1).

### Prevalence of child maltreatment types, by age

The proportions of respondents aged 65 years or more who reported maltreatment during childhood were smaller than for other age groups, with the exception of sexual abuse. For other age groups, consistent relationships between age group and physical abuse, emotional abuse, neglect, and exposure to domestic violence were not evident (Box 1, Box 2).

Box 2 Overview of reported prevalence of maltreatment during childhood (with 95% confidence intervals), by maltreatment type*

**Figure:**
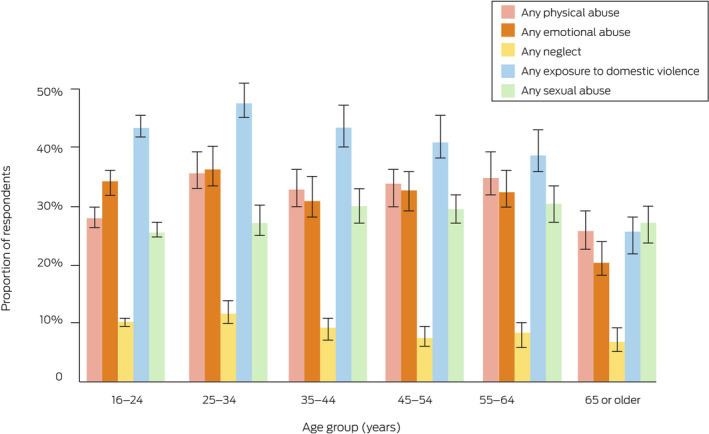
* The raw numbers underlying the listed proportions are included in the Supporting Information, tables 1–5. Proportions are weighted by age group, gender, Indigenous status, country of birth (Australia or overseas), highest educational level, and residential socio‐economic status (Relative Socio‐economic Advantage and Disadvantage quintiles).^16^

The proportion of respondents aged 16–24 years who reported physical abuse (987 people, 28.2%; 95% CI, 26.6–29.9%) was smaller than for all other age groups (range, 33.2–36.0%), with the exception of people aged 65 years or more (26.0%; 95% CI, 23.0–29.0%). The proportion of respondents aged 16–24 years who reported sexual abuse (889 people, 25.7%; 95% CI, 24.1–27.3%) was slightly smaller than for other age groups (range, 27.4–30.7%), with the exception of people aged 65 years or more (Box 1, Box 2), chiefly because the proportions reporting non‐contact abuse or abuse by adult family members were smaller (Supporting Information, table 6).

The proportions of respondents aged 16–24 years who reported emotional abuse, neglect, or exposure to domestic violence were similar to those for other age groups; the proportion reporting physical abuse was smaller than for people aged 25–34 years (28.2% [95% CI, 26.6–29.9%] *v* 36.0% [95% CI, 32.7–39.3%]), and the proportion reporting sexual abuse was smaller than for people aged 35–44 years (25.7% [95% CI, 24.1–27.3%] *v* 30.3% [95% CI, 27.1–33.5%]) (Box 1, Box 2).

## Discussion

The ACMS provides the first national prevalence estimates for all five forms of child maltreatment before the age of 18 years in Australia. We found that child maltreatment has been a serious problem in Australia for many decades, and continues to be so. Substantial proportions of respondents reported physical abuse (32.0%), sexual abuse (28.5%), or emotional abuse (30.9%) during childhood, and nearly four in ten people (39.6%) reported exposure to domestic violence. Strengths of the ACMS include its large representative sample, extensive testing and validation of the adapted version of the JVQ‐R2,^22^ conceptual rigour, and comprehensiveness. Follow‐up questions indicated that maltreatment experiences were not isolated events (Supporting Information, table 7), further bolstering the robustness of our findings.

Our findings are consistent with other research indicating that maltreatment is far more prevalent than the cases known to government agencies.^32^, ^33^ State and territory‐based government child protection agencies report maltreatment in different ways, but analyses of aggregated data indicate that a mean 0.86% of people aged 0–17 years were the subjects of substantiated reports of mistreatment each year during 2015–2020, and that about 3.0% received some type of child protection.^33^ We examined a broader range of maltreatment than typically comes to the attention of these agencies, suggesting substantial unmet need for immediate assistance and support for people experiencing long term harm. Our findings are important for informing responses by health services, educational and welfare systems, and coordinated public health policy and prevention activities. Further analyses of our data — multi‐type maltreatment and risk profiles,^34^ and mental health outcomes,^35^ health risk behaviours,^36^ and health service use associated with child maltreatment^37^ — are reported in other articles in this supplement.

We assessed the five types of maltreatment across childhood to age 18 years with an established instrument supplemented by additional validated questions in an unusually comprehensive approach by international standards.^8^ Our estimated prevalence of physical abuse during childhood (32.0%) is higher than found by a global meta‐analysis (22.6%),^5^ but similar to that of a Canadian study (26.1%; 21.3% of women, 31.0% of men).^28^ Our estimate for emotional abuse (30.9%; 35.6% of women, 25.4% of men) is lower than found by a global meta‐analysis of self‐report studies (36.3%),^7^ but may be more reliable than that from a British study based on responses to a single survey question (6.9%).^9^ Our estimate for neglect (8.9%; 10.8% of women, 6.7% of men), is lower than found by the British study (16%), which used a broad range of additional, seldom used items.^9^ Our estimate for exposure to domestic violence (39.6%; 40.8% of women, 38.0% of men) is higher than found by the British study based on different survey items (23.7%).^9^

Our estimate for sexual abuse (28.5%; 37.3% of women, 18.8% of men) is similar to that of the United Kingdom study, based on similar survey items (24.1%).^9^ It is also similar to rates reported by other Australian studies: sexual abuse during childhood was reported by 35% of women in a 1994 survey,^31^ and by 25.2% of participants (30.6% of women, 19.3% of men) in the 21‐year follow‐up of a 1980s cohort study in Queensland;^32^ in a national 2000 telephone survey, non‐penetrative sexual abuse was reported by 33.6% of women and 15.9% of men, penetrative sexual abuse by 12% of women and 4% of men.^11^ Our estimated prevalence of sexual abuse by adult family members (7.8%), about one‐third of all sexual abuse, is novel but consistent with most sexual abuse being inflicted by adults and adolescents known to the child. We plan to further analyse ACMS data to determine the prevalence of sexual abuse by people within and beyond families and institutions.

In our study, larger proportions of women than of men reported neglect and emotional abuse during childhood, and twice as many women as men reported sexual abuse. The reported prevalence of physical abuse and exposure to domestic violence was similar for women and men. These findings are similar to those of American studies that found rates of emotional^18^ and sexual abuse^38^ were higher for girls, and that rates of physical abuse were similar for both sexes.^18^ A Canadian study, however, found that physical abuse was more frequently reported by men (31.0% *v* 21.3%).^28^ Taken together, our findings indicate substantial differences in prevalence by gender, and special efforts are required to prevent the sexual and emotional abuse of girls.

Reported rates of each maltreatment type were higher for gender‐diverse than other participants, but this finding should be interpreted with caution given the small number of gender‐diverse respondents. Further, we had no information about important aspects of gender‐diverse identification; for example, we cannot determine whether emotional abuse was related to parental reactions to their child's expression of their identity. These aspects will be examined in a separate analysis of the ACMS data.

Maltreatment prevalence by age group is infrequently reported, as study designs often preclude such analysis (eg, one‐off rather than serial studies). Small, recent declines in the prevalence of non‐penetrative sexual abuse of boys and of penetrative sexual abuse of boys and girls have been reported in Australia,^11^ and a decline in sexual abuse has been reported in the United States.^18^, ^38^ We found that smaller proportions of respondents aged 16–24 years reported physical abuse and, to a lesser extent, sexual abuse, but this did not apply to emotional abuse, neglect, or exposure to domestic violence. These findings suggest that the prevalence of physical and sexual abuse have recently declined, probably influenced by changes in policy, practice, social sensitisation, education, and parenting practices in Australia. Reducing the prevalence of other maltreatment types by the same means is possible.

### Limitations

The participation rate for contacted eligible people was only 14.0%, but the demographic characteristics and health behaviours of the sample were broadly similar to those of the Australian population aged 16 years or more in 2016. Statistical weighting did not markedly influence our prevalence estimates, but ensured that they were reliable for generalising to the national population. The retrospective design entails risks of recall inaccuracy, especially for events during early childhood, which may lead to underestimating prevalence. Further, some maltreatment may have been forgotten or reframed as normal,^32^ leading to underestimation of prevalence, particularly by respondents aged 65 years or more. Another limitation was that participants aged 16 or 17 years could not provide information about events to age 18 years; however, 3122 respondents in the 16–24 year age group (89%) were 18–24 years old.

### Conclusions

Physical abuse, sexual abuse, emotional abuse, and exposure to domestic violence during childhood are common in Australia. Larger proportions of women than of men reported childhood sexual abuse, emotional abuse, and neglect. Recent declines in reported physical and sexual abuse suggest that public health initiatives can reduce the prevalence of maltreatment. These declines justify policy and prevention strategies that aim to further reduce the prevalence of these maltreatment types, and support efforts to reduce emotional abuse and exposure to domestic violence, which have received less attention than physical and sexual abuse. Monitoring in further regular studies can be used to assess changes in prevalence in Australia, facilitating comparison by age group^38^ and review of the impact of policy and prevention activities.

## Open access

Open access publishing facilitated by Queensland University of Technology, as part of the Wiley – Queensland University of Technology agreement via the Council of Australian University Librarians.

## Agency roles

The NHMRC funded the ACMS. The Australian Government provided supplementary funding for several specific questions. The researchers were independent of the funders.

## Competing interests

No relevant disclosures.

## Supporting information


**Supporting Information**.
