# Supplementary material for: The prevalence of child maltreatment in Australia: findings from a national survey
Source: Med J Aust. 2023 Apr 2;218(Suppl 6):S13–8. doi: 10.5694/mja2.51873 (PMC10953347; doi:10.5694/mja2.51873)
Supplement: Supplementary file 1 — Supporting Information. [file MJA2-218-S13-s001.pdf]

The Medical Journal of Australia

Supporting Information

Supplementary results

This appendix was part of the submitted manuscript and has been peer reviewed. It is posted as supplied by the authors.

Appendix to: Mathews B, Pacella R, Scott JG, et al. The prevalence of child maltreatment in Australia: findings from a national survey. *Med J Aust* 2023; doi: 10.5694/mja2.51873.

Table 1. Weighted proportions of respondents who reported physical abuse, overall and by subdomain, by age and gender*

	Any physical abuse		Severe physical abuse		Moderate physical abuse	
**Age group**	**Number**	**Proportion (95% CI)**	**Number**	**Proportion (95% CI)**	**Number**	**Proportion (95% CI)**
All ages	2623	32.0% (30.7–33.3%)	1639	20.1% (19.0–21.2%)	1507	18.3% (17.3–19.4%)
Women	1281	31.5% (29.7–33.3%)	820	20.2% (18.6–21.7%)	721	17.4% (15.9–18.9%)
Men	1284	32.1% (30.3–33.9%)	778	19.6% (18.0–21.1%)	757	19.2% (17.7–20.7%)
Gender-diverse	58	49.9% (37.1–62.6%)	41	41% (28.3–53.8%)	29	22.3% (12.0–32.5%)
16–24 years	987	28.2% (26.6–29.9%)	634	18.8% (17.3–20.2%)	566	16.0% (14.7–17.4%)
Women	476	29.0% (26.6–31.4%)	314	19.9% (17.7–22.1%)	274	16.4% (14.4–18.3%)
Men	468	26.3% (24.0–28.5%)	293	17.0% (15.0–18.9%)	269	15.1% (13.2–17.0%)
25–34 years	349	36.0% (32.7–39.3%)	224	23.5% (20.6–26.3%)	187	18.9% (16.3–21.6%)
Women	167	37.7% (32.9–42.5%)	109	25.0% (20.7–29.3%)	87	19.3% (15.3–23.2%)
Men	176	34.0% (29.5–38.5%)	110	21.4% (17.5–25.3%)	97	18.4% (14.8–22.1%)
35–44 years	328	33.2% (30.0–36.5%)	199	21.4% (18.5–24.3%)	178	17.7% (15.1–20.3%)
Women	165	33.3% (28.8–37.9%)	105	22.6% (18.5–26.7%)	89	16.7% (13.2–20.1%)
Men	159	32.8% (28.1–37.6%)	90	20% (16–24%)	87	18.5% (14.6–22.5%)
45–54 years	340	34.2% (30.9–37.5%)	205	21.0% (18.2–23.8%)	219	22.8% (19.9–25.7%)
Women	170	32.8% (28.2–37.3%)	108	21.3% (17.3–25.3%)	103	20.6% (16.7–24.5%)
Men	170	36.0% (31.2–40.7%)	97	21% (17–25%)	116	25.3% (20.9–29.6%)
55–64 years	349	35.2% (31.9–38.4%)	222	22.3% (19.5–25.2%)	204	20.5% (17.7–23.3%)
Women	170	34.4% (29.8–39.1%)	108	21.8% (17.8–25.8%)	98	19.6% (15.7–23.5%)
Men	176	35.5% (30.8–40.2%)	111	22.4% (18.3–26.4%)	105	21.5% (17.5–25.5%)
65 years or older	270	26.0% (23.0–29.0%)	155	14.6% (12.2–17.0%)	153	14.8% (12.4–17.3%)
Women	133	24.1% (20.0–28.1%)	76	13% (9.8–15.9%)	70	13.5% (10.1–16.8%)
Men	135	28.2% (23.6–32.7%)	77	16% (12.6–20.1%)	83	16.7% (12.9–20.5%)

CI = confidence interval. Proportions are weighted by age group, gender, Indigenous status, country of birth (Australia or overseas), highest educational level, and residential socio-economic status (Relative Socio-economic Advantage and Disadvantage quintiles).

Table 2. Weighted proportions of respondents who reported emotional abuse, overall and by subdomain, by age and gender*

	Any emotional abuse		Hostile interaction/denigration		Rejection		Emotional unavailability	
**Age group**	**Number**	**Proportion (95% CI)**	**Number**	**Proportion (95% CI)**	**Number**	**Proportion (95% CI)**	**Number**	**Proportion (95% CI)**
All ages	2743	30.9% (29.7–32.2%)	2154	23.8% (22.6–24.9%)	810	8.8% (8.0–9.6%)	1870	21.6% (20.5–22.7%)
Women	1573	35.6% (33.8–37.4%)	1275	28.4% (26.7–30.1%)	475	10.2% (9.1–11.4%)	1097	25.1% (23.4–26.7%)
Men	1088	25.4% (23.7–27.1%)	805	18.3% (16.8–19.7%)	303	7.0% (6.0–7.9%)	716	17.5% (16.0–18.9%)
Gender diverse	82	58.3% (45.4–71.3%)	74	55.2% (42.3–68.0%)	32	25.0% (14.3–35.6%)	57	41.4% (29.0–53.7%)
16–24 years	1199	34.6% (32.8–36.3%)	962	27.7% (26.0–29.3%)	386	11.2% (10.1–12.4%)	771	22.2% (20.7–23.7%)
Women	666	40.5% (37.9–43.1%)	551	33.8% (31.2–36.3%)	226	13.7% (11.9–15.5%)	452	27.2% (24.9–29.6%)
Men	470	26.9% (24.6–29.3%)	356	20.0% (17.9–22.1%)	136	7.8% (6.4–9.2%)	275	15.9% (13.9–17.8%)
25–34 years	356	36.7% (33.5–40.0%)	284	28.9% (25.8–32.0%)	116	11.8% (9.7–14.0%)	244	25.6% (22.6–28.5%)
Women	196	42.7% (37.9–47.6%)	160	34.1% (29.5–38.8%)	71	15% (12–19%)	137	30.2% (25.6–34.7%)
Men	150	29.8% (25.4–34.1%)	114	22.5% (18.5–26.5%)	41	8.0% (5.4–10%)	101	20.4% (16.5–24.3%)
35–44 years	307	31.2% (28.0–34.4%)	249	24.9% (21.9–27.9%)	86	9.0% (7.0–11%)	199	20.4% (17.5–23.2%)
Women	192	37.8% (33.2–42.5%)	163	32.0% (24.5–36.5%)	50	10% (7.1–13%)	126	24.9% (20.7–29.0%)
Men	110	24.1% (19.7–28.5%)	81	17.2% (13.4–21.1%)	33	7.5% (4.8–10%)	70	15.6% (11.8–19.5%)
45–54 years	337	33.0% (29.7–36.2%)	266	26.2% (23.1–29.2%)	89	9.5% (7.5–12%)	251	24.8% (21.8–27.8%)
Women	200	37.5% (32.9–42.2%)	158	30.1% (25.7–34.6%)	51	11.1% (7.9–14%)	147	28.5% (24.2–32.9%)
Men	136	28.6% (24.1–33.1%)	107	22.3% (18.2–26.5%)	38	8.0% (5.4–11%)	103	21.2% (17.1–25.2%)
55–64 years	335	32.7% (29.5–35.9%)	244	23.4% (20.5–26.3%)	93	8.5% (6.6–10%)	257	24.6% (21.7–27.5%)
Women	194	37.4% (32.7–42.1%)	152	29.3% (24.9–33.7%)	54	9.4% (6.6–12%)	145	27.2% (22.9–31.5%)
Men	139	27.6% (23.3–31.8%)	90	16.8% (13.4–20.3%)	38	7.4% (5.0–9.8%)	110	21.7% (17.8–25.6%)
65 years or older	209	20.5% (17.7–23.3%)	149	14.3% (11.9–16.7%)	40	4.1% (2.7–5.6%)	148	14.2% (11.8–16.6%)
Women	125	23.2% (19.2–27.3%)	91	16.5% (13.0–20.0%)	23	4.6% (2.6–6.6%)	90	16.4% (12.9–19.9%)
Men	83	17.0% (13.2–20.8%)	57	11.4% (8.2–14.6%)	17	3.6% (1.6–5.6%)	57	11.2% (8.0–14.4%)

CI = confidence interval. Proportions are weighted by age group, gender, Indigenous status, country of birth (Australia or overseas), highest educational level, and residential socio-economic status (Relative Socio-economic Advantage and Disadvantage quintiles).

Table 3. Weighted proportions of respondents who reported neglect, overall and by subdomain, by age and gender*

Age group	Any neglect		Environmental		Physical		Medical	
	Number	Proportion (95% CI)	Number	Proportion (95% CI)	Number	Proportion (95% CI)	Number	Proportion (95% CI)
All ages	759	8.9% (8.1–9.7%)	361	3.9% (3.4–4.4%)	335	4.1% (3.6–4.7%)	402	4.8% (4.2–5.4%)
Women	449	10.8% (9.5–12.0%)	207	4.5% (3.7–5.3%)	194	4.9% (4.0–5.7%)	261	6.4% (5.4–7.4%)
Men	276	6.7% (5.7–7.6%)	138	3.2% (2.5–3.9%)	125	3.2% (2.5–3.9%)	118	2.8% (2.1–3.4%)
Gender diverse	34	26% (15–38%)	16	6.3% (2.7–10%)	16	12% (3.8–20%)	23	22% (11–33%)
16–24 years	353	10.3% (9.2–11.4%)	181	5.2% (4.4–6.0%)	138	4.0% (3.3–4.8%)	187	5.6% (4.7–6.4%)
Women	201	12.5% (10.7–14.2%)	97	6.0% (4.8–7.3%)	73	4.7% (3.5–5.8%)	118	7.3% (6.0–8.7%)
Men	126	7.2% (5.8–8.5%)	68	3.7% (2.8–4.6%)	52	2.9% (2.1–3.7%)	54	3.2% (2.2–4.2%)
25–34 years	108	11.7% (9.5–14.0%)	57	5.8% (4.2–7.4%)	51	5.5% (3.9–7.1%)	56	6.3% (4.5–8.0%)
Women	63	15% (11–18%)	32	6.7% (4.3–9.1%)	30	7.1% (4.4–9.7%)	37	8.8% (5.8–12%)
Men	41	8.3% (5.6–11%)	25	5.1% (3.0–7.2%)	19	3.6% (1.9–5.3%)	15	3.0% (1.4–4.7%)
35–44 years	84	9.3% (7.2–11%)	36	3.8% (2.5–5.1%)	43	4.5% (3.0–6.0%)	52	5.8% (4.1–7.5%)
Women	57	13% (9.3–16%)	29	5.9% (3.6–8.2%)	30	6.1% (3.7–8.5%)	37	8.6% (5.6–12%)
Men	26	5.7% (3.3–8.0%)	7	1.7% (0.4–2.9%)	13	3.0% (1.2–4.8%)	14	2.9% (1.3–4.5%)
45–54 years	68	7.5% (5.5–9.4%)	28	2.8% (1.7–3.9%)	32	3.6% (2.3–4.9%)	36	4.2% (2.6–5.7%)
Women	40	8.1% (5.3–11%)	17	2.7% (1.3–4.0%)	21	4.3% (2.3–6.2%)	22	5.1% (2.7–7.5%)
Men	27	6.8% (4.1–9.4%)	11	2.9% (1.1–4.8%)	11	2.9% (1.1–4.6%)	13	3.2% (1.3–5.0%)
55–64 years	83	8.4% (6.5–10%)	31	3.2% (2.0–4.5%)	39	3.8% (2.5–5.1%)	41	4.0% (2.7–5.3%)
Women	50	10% (7.1–13%)	17	3.5% (1.7–5.4%)	20	3.8% (2.0–5.7%)	26	5.2% (3.1–7.4%)
Men	32	6.6% (4.2–9.0%)	14	2.9% (1.3–4.6%)	18	3.7% (1.9–5.5%)	14	2.6% (1.1–4.0%)
65 years or older	63	6.8% (5.0–8.6%)	28	2.8% (1.6–3.9%)	32	3.3% (2.0–4.5%)	30	3.2% (2.0–4.5%)
Women	38	7.6% (5.0–10%)	15	2.7% (1.2–4.3%)	20	3.5% (1.8–5.2%)	21	4.2% (2.2–6.1%)
Men	24	5.7% (3.3–8.0%)	13	2.8% (1.2–4.5%)	12	3.0% (1.2–4.8%)	8	1.9% (0.5–3.2%)

CI = confidence interval. Proportions are weighted by age group, gender, Indigenous status, country of birth (Australia or overseas), highest educational level, and residential socio-economic status (Relative Socio-economic Advantage and Disadvantage quintiles).

Table 4. Weighted proportions of respondents who reported exposure to domestic violence, overall and by subdomain, by age and gender*

Age group	Any exposure to domestic violence		Physical violence between parents		Serious threats between parents		Parent damaging property/pets		Parental intimidation or control	
	Number	Proportion (95% CI)	Number	Proportion (95% CI)	Number	Proportion (95% CI)	Number	Proportion (95% CI)	Number	Proportion (95% CI)
All ages	3487	39.6% (38.3–40.9%)	1606	19.9% (18.8–20.9%)	1310	15.5% (14.5–16.4%)	2612	28.7% (27.5–29.9%)	1921	22.0% (20.9–23.1%)
Women	1790	40.8% (38.9–42.6%)	855	21.4% (19.8–22.9%)	711	16.9% (15.5–18.3%)	1318	28.7% (26.9–30.4%)	1085	24.9% (23.2–26.5%)
Men	1619	38.0% (36.1–39.9%)	713	18.0% (16.5–19.5%)	562	13.7% (12.4–15.1%)	1231	28.5% (26.7–30.2%)	792	18.7% (17.2–20.2%)
Gender diverse	78	58.2% (45.3–71.1%)	38	33.9% (21.8–45.9%)	37	28.1% (16.8–39.4%)	63	44.7% (32.2–57.1%)	44	33.3% (21.4–45.3%)
16–24 years	1509	43.8% (42.0–45.6%)	598	17.6% (16.2–19.0%)	544	15.9% (14.5–17.2%)	1212	35.3% (33.5–37.1%)	792	22.5% (21.0–24.1%)
Women	755	45.8% (43.2–48.5%)	317	19.6% (17.5–21.7%)	284	17.4% (15.4–19.5%)	608	37.2% (34.6–39.8)	443	26.6% (24.2–28.9%)
Men	697	40.8% (38.2–43.3%)	259	15.6% (13.6–17.5%)	231	13.4% (11.6–15.2%)	557	32.4% (30.0–34.9%)	317	18.0% (16.0–20.1%)
25–34 years	474	48.1% (44.7–51.5%)	237	23.9% (21.0–26.8%)	188	18.9% (16.3–21.6%)	378	38.5% (35.2–41.8%)	249	25.4% (22.4–28.3%)
Women	228	49.0% (44.1–54.0%)	116	25.5% (21.2–29.8%)	102	21.5% (17.5–25.5%)	179	39.1% (34.3–43.9%)	136	29.1% (24.6–33.6%)
Men	238	47.0% (42.2–51.7%)	115	21.8% (17.9–25.7%)	82	16% (12–19%)	192	37.5% (32.9–42.1%)	107	21.0% (17.1–25.0%)
35–44 years	436	43.9% (40.4–47.3%)	198	20.3% (17.5–23.1%)	168	17.9% (15.2–20.6%)	340	35.2% (31.9–38.5%)	217	21.0% (18.3–23.8%)
Women	240	45.7% (40.9–50.5%)	110	21.9% (18.0–25.9%)	102	21.0% (17.1–24.9%)	179	34.9% (30.4–39.5%)	137	26.0% (21.9–30.2%)
Men	189	41.5% (36.5–46.5%)	83	18% (14–22%)	64	15% (11–18%)	155	34.9% (30.0–39.8%)	78	16% (12–20%)
45–54 years	424	41.3% (38.0–44.7%)	215	21.4% (18.6–24.2%)	154	14.8% (12.4–17.2%)	290	27.7% (24.6–30.8%)	254	24.5% (21.5–27.4%)
Women	225	42.4% (37.6–47.1%)	119	23.7% (19.5–27.9%)	87	16% (12–20%)	146	26.3% (22.1–30.5%)	145	27.3% (23.0–31.6%)
Men	196	40.1% (35.2–45.0%)	93	18.7% (14.9–22.5%)	67	13.7% (10.3–17.1%)	143	29.3% (24.8–33.9%)	108	21.8% (17.7–25.8%)

Age group	Any exposure to domestic violence		Physical violence between parents		Serious threats between parents		Parent damaging property/pets		Parental intimidation or control	
	Number	Proportion (95% CI)	Number	Proportion (95% CI)	Number	Proportion (95% CI)	Number	Proportion (95% CI)	Number	Proportion (95% CI)
55–64 years	389	39.1% (35.7–42.4%)	216	22.5% (19.6–25.4%)	160	16.7% (14.1–19.3%)	257	25.9% (22.9–28.9%)	248	25.1% (22.1–28.0%)
Women	199	39.9% (35.2–44.6%)	117	24.5% (20.3–28.7%)	84	17.1% (13.4–20.7%)	132	26.2% (22.0–30.5%)	134	27.1% (22.8–31.4%)
Men	188	38.2% (33.4–43.0%)	97	20.2% (16.2–24.2%)	75	16.2% (12.5–19.9%)	123	25.5% (21.2–29.7%)	112	22.8% (18.7–26.8%)
65 years or older	255	25.0% (22.1–28.0%)	142	14.4% (11.9–16.8%)	96	9.8% (7.7–12%)	135	13.3% (11.0–15.7%)	161	15.4% (12.9–17.9%)
Women	143	27.3% (23.1–31.5%)	76	15.1% (11.7–18.6%)	52	10.4% (7.5–13.3%)	74	14.2% (11.0–17.5%)	90	16.7% (13.2–20.2%)
Men	111	22.2% (18.1–26.4%)	66	13.6% (10.1–17.1%)	43	8.9% (6.0–11.8%)	61	12.4% (9.0–15.8%)	70	13.6% (10.2–17.0%)

CI = confidence interval. Proportions are weighted by age group, gender, Indigenous status, country of birth (Australia or overseas), highest educational level, and residential socio-economic status (Relative Socio-economic Advantage and Disadvantage quintiles).

Table 5. Weighted proportions of respondents who reported sexual abuse (by any person), overall and by subdomain, by age and gender*

Age group	Any sexual abuse		Contact sexual abuse: Any contact sexual abuse		Contact sexual abuse: Contact abuse short of intercourse (touching)		Contact sexual abuse: Attempted forced intercourse		Contact sexual abuse: Forced intercourse		Non-contact sexual abuse: Exposure or voyeurism	
	Number	Proportion (95% CI)	Number	Proportion (95% CI)	Number	Proportion (95% CI)	Number	Proportion (95% CI)	Number	Proportion (95% CI)	Number	Proportion (95% CI)
All ages	2348	28.5% (27.3–29.8%)	1960	23.7% (22.6–24.9%)	1525	18.9% (17.9–20.0%)	1201	13.8% (12.9–14.7%)	717	8.7% (7.9–9.4%)	1443	18.1% (17.0–19.1%)
Women	1536	37.3% (35.5–39.2%)	1336	32.4% (30.6–34.2%)	1017	25.5% (23.8–27.2%)	861	19.4% (17.9–21.0%)	519	12.5% (11.2–13.8%)	911	22.9% (21.3–24.5%)
Men	739	18.8% (17.3–20.3%)	563	14.2% (12.9–15.6%)	452	11.6% (10.4–12.9%)	296	7.4% (6.4–8.5%)	167	4.3% (3.5–5.2%)	476	12.6% (11.3–13.9%)
Gender diverse	73	51.9% (39.1–64.7%)	61	45.1% (32.5–57.6%)	56	41.0% (28.7–53.3%)	44	31.7% (20.3–43.0%)	31	23.4% (13.1–33.6%)	56	38.6% (26.4–50.7%)
16–24 years	889	25.7% (24.1–27.3%)	759	22.0% (20.4–23.5%)	555	16.2% (14.9–17.6%)	525	15.2% (13.9–16.5%)	303	8.7% (7.7–9.7%)	506	15.0% (13.7–16.4%)
Women	589	35.2% (32.7–37.8%)	532	32.0% (29.5–34.4%)	385	23.6% (21.3–25.8%)	383	23.1% (20.9–25.3%)	219	13.1% (11.3–14.9%)	318	19.7% (17.6–21.8%)
Men	244	14.5% (12.6–16.5%)	181	10.6% (8.9–12.2%)	128	7.4% (6.0–8.8%)	109	6.3% (5.1–7.6%)	62	3.6% (2.6–4.6%)	144	8.7% (7.1–10.3%)
25–34 years	264	27.4% (24.4–30.5%)	230	24.2% (21.3–27.2%)	180	18.6% (16.0–21.3%)	143	15.1% (12.6–17.6%)	99	10.8% (8.6–13.0%)	162	17.0% (14.4–19.5%)
Women	170	37.6% (32.8–42.4%)	157	35.2% (30.5–40.0%)	122	27.1% (22.7–31.5%)	105	23.1% (18.9–27.3%)	70	16% (13–20%)	100	22.6% (18.4–26.8%)
Men	88	16.9% (13.4–20.5%)	67	12.8% (9.7–16.0%)	52	9.6% (6.8–12.3%)	34	6.8% (4.5–9.2%)	26	5.1% (3.0–7.3%)	58	10.9% (8.0–14%)
35–44 years	304	30.3% (27.1–33.5%)	233	23.5% (20.6–26.4%)	195	19.8% (17.0–22.5%)	109	11.6% (9.3–13.8%)	64	7.2% (5.4–9.1%)	205	20.4% (17.6–23.2%)
Women	203	40.1% (35.3–44.8%)	157	32.0% (27.5–36.6%)	129	26.8% (22.5–31.2%)	78	16.9% (13.1–20.7%)	46	10% (7.3–13%)	139	28.0% (23.6–32.3%)
Men	96	20.2% (16.2–24.2%)	72	14.6% (11.1–18.1%)	63	12.5% (9.3–15.7%)	27	5.7% (3.3–8.0%)	15	3.7% (1.6–5.8%)	63	12.7% (9.4–16%)
45–54 years	304	29.8% (26.7–32.9%)	256	25.3% (22.3–28.2%)	208	21.0% (18.2–23.8%)	165	16.7% (14.1–19.2%)	98	10.2% (8.1–12%)	209	20.9% (18.1–23.7%)
Women	200	38.8% (34.1–43.5%)	173	34.0% (29.5–38.6%)	143	29.0% (24.6–33.3%)	106	21.3% (17.3–25.2%)	69	14% (11–18%)	135	26.5% (22.3–30.8%)

Age group	Any sexual abuse		Contact sexual abuse								Non-contact sexual abuse	
			Any contact sexual abuse		Contact abuse short of intercourse (touching)		Attempted forced intercourse		Forced intercourse		Exposure or voyeurism	
	Number	Proportion (95% CI)	Number	Proportion (95% CI)	Number	Proportion (95% CI)	Number	Proportion (95% CI)	Number	Proportion (95% CI)	Number	Proportion (95% CI)
Men	102	20.5% (16.6–24.5%)	81	16.2% (12.7–19.7%)	63	12.8% (9.6–16.0%)	57	11.8% (8.7–15.0%)	28	5.8% (3.5–8.1%)	73	15.2% (11.7–18.8%)
55–64 years	315	30.7% (27.6–33.9%)	263	25.5% (22.6–28.5%)	209	20.1% (17.4–22.8%)	160	15.2% (12.8–17.6%)	92	8.6% (6.7–10.4%)	203	19.3% (16.6–21.9%)
Women	203	40.2% (35.5–45.0%)	177	35.0% (30.3–39.6%)	131	26.0% (21.8–30.2%)	122	23.0% (19.0–27.0%)	71	13.3% (10.1–16.5%)	129	24.4% (20.4–28.5%)
Men	109	19.8% (16.1–23.6%)	84	15.2% (11.8–18.5%)	76	13.5% (10.4–16.7%)	37	6.8% (4.4–9.1%)	20	3.2% (1.7–4.8%)	71	13.0% (9.9–16.1%)
65 years or older	272	27.4% (24.3–30.5%)	219	22.3% (19.4–25.2%)	178	18.0% (15.3–20.7%)	99	10.3% (8.2–12%)	61	6.9% (5.1–8.7%)	158	16.3% (13.7–18.9%)
Women	171	33.4% (28.9–37.9%)	140	27.8% (23.5–32.1%)	107	21.5% (17.6–25.5%)	67	12.8% (9.7–16.0%)	44	8.8% (6.1–11.5%)	90	17.8% (14.2–21.5%)
Men	100	20.1% (16.0–24.1%)	78	15.5% (11.8–19.1%)	70	13.5% (10.0–16.9%)	32	7.2% (4.6–9.9%)	16	4.3% (2.0–6.6%)	67	14.3% (10.7–18.0%)

CI = confidence interval. Proportions are weighted by age group, gender, Indigenous status, country of birth (Australia or overseas), highest educational level, and residential socio-economic status (Relative Socio-economic Advantage and Disadvantage quintiles).

Table 6. Weighted proportions of respondents who reported sexual abuse (by adult family member), overall and by subdomain, by age and gender*

Age group			Contact sexual abuse								Non-contact sexual abuse	
	Any sexual abuse		Any contact sexual abuse		Contact abuse short of intercourse (touching)		Attempted forced intercourse		Forced intercourse		Exposure or voyeurism	
	Number	Proportion (95% CI)	Number	Proportion (95% CI)	Number	Proportion (95% CI)	Number	Proportion (95% CI)	Number	Proportion (95% CI)	Number	Proportion (95% CI)
All ages	551	7.8% (7.0–8.5%)	464	6.7% (6.0–7.5%)	423	6.1% (5.4–6.8%)	214	3.1% (2.6–3.6%)	135	1.9% (1.5–2.3%)	379	5.2% (4.6–5.9%)
Women	407	11.9% (10.6–13.2%)	351	10.5% (9.3–11.8%)	323	9.7% (8.5–10.9%)	156	4.5% (3.7–5.4%)	96	2.7% (2.0–3.3%)	274	7.7% (6.7–8.8%)
Men	122	3.2% (2.5–3.9%)	97	2.6% (1.9–3.2%)	86	2.2% (1.7–2.8%)	54	1.7% (1.1–2.2%)	34	1.1% (0.7–1.5%)	87	2.5% (1.8–3.1%)
Gender diverse	22	17.3% (7.4–27.1%)	16	15.1% (5.4–24.8%)	14	12.3% (3.2–21.3%)	4	3.7% (0.0–8.3%)	5	6.0% (0.0–12.7%)	18	13.8% (4.6–23.0%)
16–24 years	151	4.4% (3.7–5.2%)	130	3.9% (3.2–4.6%)	119	3.5% (2.9–4.2%)	59	1.8% (1.3–2.2%)	40	1.4% (0.9–1.8%)	103	2.9% (2.3–3.5%)
Women	101	6.4% (5.1–7.7%)	91	5.8% (4.6–7.1%)	83	5.3% (4.2–6.5%)	47	3.0% (2.1–4.0%)	30	2.0% (1.3–2.8%)	70	4.4% (3.3–5.5%)
Men	34	1.8% (1.1–2.6%)	28	1.6% (0.9–2.3%)	26	1.5% (0.8–2.2%)	10	0.5% (0.2–0.8%)	7	0.5% (0.0–1.1%)	20	1.0% (0.5–1.4%)
25–34 years	71	7.3% (5.5–9.0%)	61	6.5% (4.8–8.2%)	55	5.8% (4.2–7.5%)	33	3.7% (2.4–5.0%)	22	2.3% (1.3–3.3%)	53	5.4% (3.8–6.9%)
Women	49	11.0% (7.8–14.2%)	44	10.3% (7.2–13.4%)	39	9.2% (6.2–12.1%)	22	5.5% (3.1–7.9%)	15	3.4% (1.6–5.1%)	37	8.2% (5.4–11.0%)
Men	20	3.3% (1.8–4.8%)	15	2.4% (1.1–3.7%)	15	2.4% (1.1–3.7%)	10	1.9% (0.7–3.1%)	7	1.4% (0.3–2.4%)	15	2.4% (1.1–3.7%)
35–44 years	82	8.7% (6.7–10.7%)	58	6.4% (4.7–8.2%)	52	5.8% (4.1–7.5%)	23	2.7% (1.6–3.9%)	11	1.3% (0.5–2.1%)	59	6.2% (4.5–7.9%)
Women	68	14.7% (11.1–18.2%)	50	11.4% (8.1–14.6%)	45	10.3% (7.2–13.4%)	19	4.7% (2.5–6.9%)	8	2.0% (0.6–3.5%)	49	10.5% (7.5–13.5%)
Men	13	2.8% (1.0–4.5%)	8	1.6% (0.4–2.8%)	7	1.4% (0.3–2.6%)	4	0.8% (0.0–1.7%)	3	0.5% (0.0–1.2%)	9	2.0% (0.5–3.5%)
45–54 years	93	9.4% (7.4–11%)	82	8.5% (6.6–10.5%)	75	7.8% (5.9–9.6%)	43	4.8% (3.3–6.3%)	23	2.7% (1.5–3.9%)	63	6.6% (4.9–8.3%)
Women	70	14.2% (10.8–17.6%)	60	12.6% (9.3–15.8%)	58	11.9% (8.8–15.1%)	27	6.2% (3.8–8.7%)	16	4.0% (1.9–6.1%)	47	9.7% (6.8–12.6%)

Age group	Any sexual abuse		Contact sexual abuse								Non-contact sexual abuse	
			Any contact sexual abuse		Contact abuse short of intercourse (touching)		Attempted forced intercourse		Forced intercourse		Exposure or voyeurism	
	Number	Proportion (95% CI)	Number	Proportion (95% CI)	Number	Proportion (95% CI)	Number	Proportion (95% CI)	Number	Proportion (95% CI)	Number	Proportion (95% CI)
Men	23	4.7% (2.7–6.7%)	22	4.5% (2.6–6.5%)	17	3.6% (1.8–5.4%)	16	3.4% (1.6–5.1%)	7	1.4% (0.3–2.6%)	16	3.5% (1.7–5.2%)
55–64 years	81	8.0% (6.2–9.9%)	69	7.1% (5.3–8.9%)	64	6.4% (4.7–8.1%)	34	3.2% (2.0–4.4%)	24	2.1% (1.2–3.0%)	61	5.8% (4.2–7.3%)
Women	62	12.6% (9.3–15.8%)	54	11% (8.3–14.5%)	51	10% (7.5–13.4%)	25	4.9% (2.8–7.0%)	18	3.3% (1.7–5.0%)	45	8.8% (6.1–11.5%)
Men	17	2.8% (1.4–4.3%)	13	2.1% (0.9–3.4%)	11	1.6% (0.6–2.7%)	8	1.3% (0.3–2%)	5	0.6% (0.1–1.1%)	14	2.2% (0.9–3.4%)
65 years or older	73	8.2% (6.2–10.1%)	64	7.4% (5.5–9.3%)	58	6.9% (5.0–8.7%)	22	2.5% (1.4–3.7%)	15	1.8% (0.9–2.8%)	40	4.5% (3.0–6.0%)
Women	57	11.6% (8.6–14.7%)	52	11% (7.9–13.9%)	47	10.0% (7.1–12.9%)	16	3.1% (1.4–4.7%)	9	1.6% (0.5–2.7%)	26	5.2% (3.1–7.3%)
Men	15	3.7% (1.7–5.7%)	11	2.9% (1.1–4.8%)	10	2.7% (0.9–4.5%)	6	1.9% (0.3–3.5%)	5	1.8% (0.2–3.4%)	13	3.4% (1.4–5.4%)

CI = confidence interval. Proportions are weighted by age group, gender, Indigenous status, country of birth (Australia or overseas), highest educational level, and residential socio-economic status (Relative Socio-economic Advantage and Disadvantage quintiles).

Table 7. Reported frequency or period over which child maltreatment was experienced: weighted proportions of respondents*

	Number of incidents					
**Maltreatment type/subdomain**	**1**	**2–5**	**6–10**	**11–50**	**> 50**	**Median (IQR)**
**Any physical abuse: number**	295	698	470	593	449	
Proportion (95% CI)	11.7% (10.1–13.3%)	26.5% (24.3–28.7%)	19.1% (17.1–21.0%)	23.6% (21.5–25.7%)	19.1% (17.1–21.1%)	9.5 (7.8–11.1)
Moderate physical abuse: number	209	464	262	355	282	
Proportion (95% CI)	12.6% (10.6–14.6%)	27.8% (25.0–30.6%)	17.2% (14.8–19.5%)	22.6% (20.0–25.2%)	19.8% (17.2–22.3%)	9.4 (7.5–11.3)
Severe physical abuse: number	173	462	290	320	167	
Proportion (95% CI)	12.2% (10.0–14.4%)	31.7% (28.6–34.8%)	20.1% (17.5–22.7%)	23.4% (20.6–26.2%)	12.6% (10.4–14.8%)	9.1 (7.2–10.9)
**Any sexual abuse (any person): number**	512	834	298	416	234	
Proportion (95% CI)	21.9% (19.8–24.0%)	35.8% (33.4–38.4%)	11.9% (10.3–13.6%)	18.7% (16.7–20.8%)	11.6% (9.9–13.4%)	3.5 (3.1–3.8)
Any contact sexual abuse: number	473	715	253	291	176	
Proportion (95% CI)	24.0% (21.6–26.4%)	36.2% (33.5–39.0%)	13.6% (11.6–15.6%)	15.8% (13.7–18.0%)	10.3% (8.4–12.2%)	2.9 (2.7–3.2)
Any non-contact sexual abuse: number	450	497	163	183	85	
Proportion (95% CI)	30.6% (27.7–33.6%)	35.4% (32.2–38.6%)	12.2% (10.1–14.4%)	14.5% (12.1–17.0%)	7.2% (5.4–9.0%)	2.4 (2.1–2.8)
**Any exposure to domestic violence: number**	376	868	404	738	993	
Proportion (95% CI)	11.0% (9.7–12.4%)	24.7% (22.8–26.6%)	11.5% (10.1–12.8%)	21.0% (19.2–22.8%)	31.9% (29.9–34.0%)	11.8 (10.4–13.2)
Physical violence between parents: number	251	551	229	324	193	
Proportion (95% CI)	16.0% (13.7–18.2%)	32.8% (29.9–35.8%)	15.0% (12.8–17.4%)	21.5% (18.9–24.2%)	14.7% (12.4–17.2%)	5.5 (4.4–6.6)
Serious threats: number	151	436	205	275	190	
Proportion (95% CI)	11.9% (9.6–14.4%)	31.6% (28.4–34.8%)	17.3% (14.7–20.0%)	21.9% (19.0–24.8%)	17.2% (14.5–20.0%)	9.2 (7.1–11.3)
Damage property or pets: number	484	1040	378	445	182	
Proportion (95% CI)	19.5% (17.5–21.6%)	38.8% (36.3–41.2%)	14.9% (13.1–16.8%)	18.0% (16.0–20.0%)	8.9% (7.5–10.4%)	4.5 (4.0–4.9)
Intimidation and control: number	74	404	270	506	526	
Proportion (95% CI)	3.2% (2.2–4.2%)	21.1% (18.7–23.4%)	14.6% (12.6–16.8%)	27.3% (24.7–30.0%)	33.8% (30.9–36.6%)	25.1 (15.3–34.8)

Maltreatment type/subdomain	Period over which maltreatment was experienced			
**Emotional abuse**	**Days**	**Weeks**	**Months**	**Years**
Hostility/denigration: number	180	106	230	1818
Proportion (95% CI)	7.6% (6.1–9.0%)	4.3% (3.3–5.3%)	8.8% (7.3–10.2%)	79.4% (77.3–81.5%)
Rejection: number	144	39	91	680
Proportion (95% CI)	13.5% (10.8–16.2%)	3.2% (1.9–4.6%)	7.2% (5.1–9.4%)	76.1% (72.7–79.6%)
Emotional unavailability: number	145	77	165	1628
Proportion (95% CI)	5.6% (4.4–6.8%)	3.1% (2.2–4.2%)	6.1% (4.9–7.4%)	85.2% (83.3–87.2%)
**Neglect**				
Environmental neglect: number	27	24	50	287
Proportion (95% CI)	8.1% (4.3–12%)	4.8% (2.2–7.4%)	10% (6.4–14%)	77.1% (71.7–82.6%)
Physical neglect: number	23	18	31	286
Proportion (95% CI)	4.9% (2.4–7.4%)	6.3% (2.5–10%)	6.7% (3.2–10%)	82.2% (76.9–87.4%)
Medical neglect: number	89	34	48	320
Proportion (95% CI)	18% (14–22%)	5.8% (3.1–8.4%)	9.6% (6.1–13%)	66.8% (61.5–72.2%)

CI = confidence interval; IQR = interquartile range.

* Proportions (denominator: total participants reporting any level of the maltreatment type/subdomain) refer are weighted by age group, gender, Indigenous status, country of birth (Australia or overseas), highest educational level, and residential socio-economic status (Relative Socio-economic Advantage and Disadvantage quintiles).
